# Supplementary material for: Identification of prognostic genes associated with tolerogenic dendritic cells in gastric cancer based on transcriptomic data
Source: PLoS One. 2026 Mar 5;21(3):e0343688. doi: 10.1371/journal.pone.0343688 (PMC12962506; doi:10.1371/journal.pone.0343688)
Supplement: Attachment — Submitted filename: Response to Reviewers.docx [file pone.0343688.s008.docx]

Point-by-point response to the reviewer comments.

Dear Reviewers,

Thank you for your thoughtful suggestions and insights, which have benefited from the manuscript. I am looking forward to working with you to move this manuscript closer to publication in PLOS ONE.

The manuscript has been rechecked and the necessary changes have been made in accordance with your suggestions. The responses to all comments have been prepared and attached below. We have tried our best to solve the problems you proposed, and we hope that the revised manuscript is now suitable for publication in the journal PLOS ONE. If you have any questions remained about this paper, please feel free to contact us.

**Reviewers' comments:
Reviewer's Responses to Questions**

**Comments to the Author
1. Is the manuscript technically sound, and do the data support the conclusions?
The manuscript must describe a technically sound piece of scientific research with data that supports the conclusions. Experiments must have been conducted rigorously, with appropriate controls, replication, and sample sizes. The conclusions must be drawn appropriately based on the data presented.**

**Reviewer #1: Partly**

**Reviewer #2: Partly**

Re: Dear reviewers, sincerely thank the reviewers for their strict review of the research design and data quality of this paper. We have made a comprehensive improvement on the technical rigor and conclusion support: first, we supplemented the GSE183904 single cell data set analysis, and verified the expression specificity of prognostic genes at single cell resolution; secondly, the statistical analysis method is improved, and the significance threshold is corrected by multiple tests. In addition, the prudence of conclusion expression is strengthened, and the research findings and speculative conclusions are clearly distinguished. These improvements have significantly enhanced the scientific rigor of the research and the reliability of the conclusions. Thank you again for the valuable constructive opinions of experts, which plays a vital role in improving the quality of the paper.

**2. Has the statistical analysis been performed appropriately and rigorously?**

**Reviewer #1: Yes**

**Reviewer #2: No**

Re: Dear reviewers, sincerely thank the two reviewers for their review of the statistical methods in this paper. We have carried out a comprehensive verification and improvement of statistical analysis according to the recommendation: we list the statistical methods in detail; multiple comparisons have been corrected for FDR ( lines 342-354 of the manuscript ). These improvements ensure the rigor of statistical analysis and the repeatability of results. Special thanks to the review experts put forward valuable opinions, so that we can further improve the research methodology.

**3. Have the authors made all data underlying the findings in their manuscript fully available?
The** [**PLOS Data policy**](http://www.plosone.org/static/policies.action#sharing) **requires authors to make all data underlying the findings described in their manuscript fully available without restriction, with rare exception (please refer to the Data Availability Statement in the manuscript PDF file). The data should be provided as part of the manuscript or its supporting information, or deposited to a public repository. For example, in addition to summary statistics, the data points behind means, medians and variance measures should be available. If there are restrictions on publicly sharing data—e.g. participant privacy or use of data from a third party—those must be specified.**

**Reviewer #1: Yes**

**Reviewer #2: Yes**

Re: Dear reviewer, thank you for your recognition and valuable comments on this study. We fully endorse and strictly abide by the data sharing policy of PLOS journals. All the original data involved in this study are derived from public databases: TCGA-STAD data set is derived from the cancer genome map database, and gene expression data is derived from the GEO database. The access links and numbers have been clearly marked in the method section of this study. All data can be publicly available without any restrictions. We will ensure that this information is fully presented in the manuscript through the data availability statement. Thank you again for your hard work and guidance.

**4. Is the manuscript presented in an intelligible fashion and written in standard English?
PLOS ONE does not copyedit accepted manuscripts, so the language in submitted articles must be clear, correct, and unambiguous. Any typographical or grammatical errors should be corrected at revision, so please note any specific errors here.**

**Reviewer #1: Yes**

**Reviewer #2: Yes**

Re: Dear reviewers, thank you very much for your recognition of our manuscripts, You pointed out that the ' clear presentation and use of standard English ' this evaluation so that we are encouraged. We are well aware of the importance of language expression for academic communication, so we pay special attention to the accuracy and clarity of language in the process of writing. Thank you again for your affirmation and support. Your comments are of great significance to us.

**5. Review Comments to the Author
Please use the space provided to explain your answers to the questions above. You may also include additional comments for the author, including concerns about dual publication, research ethics, or publication ethics. (Please upload your review as an attachment if it exceeds 20,000 characters)**

**Reviewer #1: 1.Please improve the resolution of figure 1, 2, 5B, 7C, 7D, 9A and 9B. The quality of those figures are not good. It is very hard to identify the letters.**

Re: Dear reviewer, thank you for your valuable comments. We have fully optimized Figure 1, Figure 2, Figure 5B, Figure 7C, Figure 7D, Figure 9A and Figure 9B according to your requirements. By improving the resolution and adjusting the font format, the clarity and recognizability of the text in the figure are significantly improved. Thank you again for your careful review and guidance.

**2. It is better to separate the figure legend from the results content.**

Re: Dear reviewer, thank you for your valuable suggestions. We understand that it may be clearer to separate the annotations from the results, but unfortunately, the format of the journal requires the annotations to be included in the description of the results in the text. In order to comply with the journal submission guide, we have maintained the existing format arrangement in this revision. Thank you again for your careful review and professional guidance.

**Reviewer #2: The authors identified five prognostic genes (CXCL1, INHBA, ASCL2, RNASE1, and GPX3) associated with tolerogenic dendritic cells in gastric cancer and constructed a risk prediction model. They found that these genes were closely linked to immune cell infiltration, particularly GPX3, which showed a strong positive correlation with regulatory T cells. However, there are several major issues that need to be addressed before this manuscript can be considered for publication.
The main weakness lies in the lack of clear motivation and conclusion drawn from the analytical results. Much of the text is descriptive, making the manuscript difficult to follow. The overall writing should be substantially improved to tell a coherent story rather than merely present a series of analyses.**
Re: Dear reviewer, thank you for your important comments. We have systematically revised the full text according to the suggestions, supplemented the clear research purpose before the description of the analysis method, added the connection statement in the result part to enhance the logical coherence, and refined the significance of the key analysis results, thus strengthening the internal relationship between the various parts, so that the paper changes from a simple data presentation to a complete story as a whole. Thank you again for your valuable comments, which played a crucial role in improving the quality of the paper.

**I strongly recommend that the authors reorganize the figures into six or seven concise figures to make the narrative more cohesive—for instance, merging Figures 1 and 2. In its current form, the paper reads more like an analysis report than a research article.**
Re: Dear reviewer, thank you for your careful review. We have carefully studied and adopted your opinions, substantially reorganized and optimized the full-text charts, and integrated the original charts into eight logically coherent comprehensive charts. This adjustment significantly enhances the sense of hierarchy and narrative fluency of the results. Thank you again for your valuable guidance to improve the quality of the paper.

**In addition, I suggest including a single-cell RNA-seq dataset to evaluate the expression of these genes in tolerogenic dendritic cells. Based on these data, the authors could even construct a regulatory network of the identified genes.**

Re: Dear reviewer, thank you for your valuable suggestions. In accordance with your guidance, we have added an analysis of the GSE183904 single-cell data set and systematically evaluated the expression patterns of prognostic genes in key cell subsets such as dendritic cells ( related content is in the manuscript 304-340,548-566,774-778 ). This supplementary analysis not only verified the specificity of gene expression, but also provided single-cell evidence for elucidating its potential function in the immune microenvironment of gastric cancer. Thank you again for your insightful opinions, which plays a vital role in improving the quality of the paper.

**Figure 5 is unclear and requires further explanation—how were the proposed transcription factors validated? Also, please clarify the rationale for selecting 67 as the cutoff point for age group analysis.**

Re: Dear reviewer, thank you very much for your important questions. For the source of the transcription factors in Figure 5, we use the htfTARGET database (https://guolab.wchscu.cn/hTFtarget/ # ! / targets /) were obtained by directly using five prognostic genes for prediction, which is indeed one of the limitations of our study. The regulatory relationship of these predictions has not been experimentally verified, and we plan to improve it through experimental verification in subsequent studies ( related content is in line 785-794 of the manuscript ). The selection of 67 years old as the age grouping threshold is based on the median age of all patients in the TCGA-STAD data set, which aims to ensure the balance of sample size between groups and improve the effectiveness of statistical tests ( related content is in line 237-238 of the manuscript ). Thank you again for your valuable comments, which are essential to enhance the rigour of our research.

**Figure 1D should adopt the same color scheme as the other panels for consistency.**

Re: Dear reviewer, thank you for your valuable suggestions. We have adjusted the color scheme of Figure 1D according to your requirements so that it is completely consistent with other subgraphs, thus ensuring the unity and coordination of the visual presentation of the entire group. Thank you again for your careful review and professional guidance.

**Finally, there are numerous formatting errors throughout the manuscript, such as “Line 302 3.3” and “Line 327 3.5.” The authors should carefully proofread the entire manuscript to correct these issues.**

Re: Dear reviewer, thank you for pointing out the format problems in the manuscript in detail. We have carried out line-by-line proofreading and comprehensive correction of the full text, unified the format specification of the chapter number, and ensured that it met the requirements of the journal. Thank you again for your rigorous review and valuable guidance.
