# Supplementary material for: Identification of prognostic genes associated with tolerogenic dendritic cells in gastric cancer based on transcriptomic data
Source: PLoS One. 2026 Mar 5;21(3):e0343688. doi: 10.1371/journal.pone.0343688 (PMC12962506; doi:10.1371/journal.pone.0343688)
Supplement: Attachment — Submitted filename: Response_to_Reviewers_auresp_2.docx [file pone.0343688.s009.docx]

Point-by-point response to the reviewer comments.

Dear Reviewers,

Thank you for your thoughtful suggestions and insights, which have benefited from the manuscript. I am looking forward to working with you to move this manuscript closer to publication in PLOS ONE.

The manuscript has been rechecked and the necessary changes have been made in accordance with your suggestions. The responses to all comments have been prepared and attached below. We have tried our best to solve the problems you proposed, and we hope that the revised manuscript is now suitable for publication in the journal PLOS ONE. If you have any questions remained about this paper, please feel free to contact us.

**Comments to the Author**
**1. If the authors have adequately addressed your comments raised in a previous round of review and you feel that this manuscript is now acceptable for publication, you may indicate that here to bypass the “Comments to the Author” section, enter your conflict of interest statement in the “Confidential to Editor” section, and submit your "Accept" recommendation.**

**Reviewer #1: All comments have been addressed**

**Reviewer #2: (No Response)**

Re: Dear Reviewer # 1, Thank you for your guidance on our work and your recognition of our revisions.

Dear Reviewer # 2, Thank you for your careful guidance. We will modify and solve your problems one by one according to your opinions. Thank you for your careful review.

**2. Is the manuscript technically sound, and do the data support the conclusions?**

**The manuscript must describe a technically sound piece of scientific research with data that supports the conclusions. Experiments must have been conducted rigorously, with appropriate controls, replication, and sample sizes. The conclusions must be drawn appropriately based on the data presented.**

**Reviewer #1: Yes**

**Reviewer #2: Partly**

Re: Dear Reviewer # 1, Thank you for your recognition of the data aspect of our work.

Dear Reviewer # 2, Thank you for your careful review. We have modified the relevant expressions of data and prediction conclusions in the manuscript, and interpreted the key results more conservatively to make them more rigorous. The limitations of the manuscript ( line: 749-758 ) indicate that this study relies on predictive and hypothesis-generating research based on public databases, which needs to be verified by experiments in the future.

**3. Has the statistical analysis been performed appropriately and rigorously?**

**Reviewer #1: Yes**

**Reviewer #2: Yes**

Re: Dear reviewers, thank you for your recognition of our statistical analysis work, Thank you very much for your guidance of our work.

**4. Have the authors made all data underlying the findings in their manuscript fully available?**

**The PLOS Data policy requires authors to make all data underlying the findings described in their manuscript fully available without restriction, with rare exception (please refer to the Data Availability Statement in the manuscript PDF file). The data should be provided as part of the manuscript or its supporting information, or deposited to a public repository. For example, in addition to summary statistics, the data points behind means, medians and variance measures should be available. If there are restrictions on publicly sharing data—e.g. participant privacy or use of data from a third party—those must be specified.**

**Reviewer #1: Yes**

**Reviewer #2: Yes**

Re: Dear reviewers, thank you for your recognition of our data disclosure. We fully support and strictly abide by the data sharing policy of PLOS journals. All the raw data involved in this study were from public databases: TCGA-STAD data set was derived from the cancer genome map database, and gene expression data was derived from the GEO database. Access links and numbers have been clearly marked in the method section of this study. All data are publicly available without restrictions. We will ensure that this information is fully presented in the manuscript through the data availability statement. Thank you again for your hard work and guidance.

**5. Is the manuscript presented in an intelligible fashion and written in standard English?
PLOS ONE does not copyedit accepted manuscripts, so the language in submitted articles must be clear, correct, and unambiguous. Any typographical or grammatical errors should be corrected at revision, so please note any specific errors here.**

**Reviewer #1: Yes**

**Reviewer #2: Yes**

Re: Dear reviewers, thank you for your recognition of our article writing. We know the importance of language expression in academic communication, so we pay special attention to the accuracy and clarity of language in the process of writing. Thank you again for your affirmation and support. Your opinion is of great significance to us.

**6. Review Comments to the Author**

**Please use the space provided to explain your answers to the questions above. You may also include additional comments for the author, including concerns about dual publication, research ethics, or publication ethics. (Please upload your review as an attachment if it exceeds 20,000 characters)**

**Reviewer #1: It is accepted to be published, but the resolution of some figures still needs to be improved as it is very hard to identify. Please figure out it.**

Re: Dear reviewer, thank you for your valuable comments. We have fully optimized all the pictures according to your requirements, and improved the clarity and recognizability of the text in the picture by improving the resolution and adjusting the font format. Thank you again for your careful review and guidance.

**Reviewer #2: 1. I have no idea what changes the authors have made regarding the first two concerns — their point-by-point responses are too vague to follow. A good summary should be given in the response.**

Re：Dear reviewer, thank you for your careful guidance. I 'm sorry for not being able to clarify these two issues before, here is a more detailed description for you. First, with regard to the improvement of manuscripts, we have supplemented clear research purposes before the description of the analysis method according to your recommendations ( for example, line: 128-130 in chapter 2.2supplemented the purpose of using data set analysis to identify significantly related differential genes ).In the result section, we added connectivity statements to enhance logical coherence, and refined the significance of key analysis results ( for example, line: 387-390 in chapter 3.2 supplemented the inference of the significance of the screening results ), thus strengthening the internal relationship between the various parts. It transforms the paper from a simple data presentation to a complete storytelling whole. Secondly, for the changes in the picture, we merged Fig1A and Fig2 based on your opinions, and adjusted Fig1BCD to Supplementary Fig 1 ( Chapter 3.1-3.2). In addition, Fig 7 was revised to Supplementary Fig 2 ( Chapter 3.10 ). After your guidance, the pictures in this paper are more logical and readable, which improves the sense of hierarchy and narrative fluency of the manuscript results. Thank you again for your nuanced guidance and valuable advice.

**2. According to the single-cell analysis results, the identified prognostic genes are barely expressed in DC cells, which raises a major concern for me (the specificity of gene expression).**

Re：Dear reviewer, thank you for your valuable comments. The low absolute expression of prognostic genes you pointed out does exist, but the level of expression is not enough to directly determine whether the gene plays an important role. Therefore, we adjusted the expression in the manuscript ( line: 553-566 ) to focus on the difference in the expression of prognostic genes in dendritic cells ( DC cells ), gastric cancer group and control group. This difference can more rigorously explain the impact of these prognostic gene-related expressions in gastric cancer and further speculate that they may have potential regulatory functions. Similarly, we adjusted the description of the discussion section ( line: 749-758 ) and discussed this phenomenon more rigorously, indicating that this conclusion is predictive and needs further experimental verification.

**3. In the single-cell analysis, the authors identified a DC cell subpopulation. However, it is well known that many distinct DC subtypes have already been characterized by single-cell sequencing. Which specific DC subtype does the tolDC population described in this study correspond to, and how was it identified? Are all DC cells in this dataset considered tolDCs?**

Re：Dear reviewer, thank you for your careful review. The tolDC data we used are all from public datasets, and these datasets are all data of moDC subpopulation cells, so the tolDC population described in this study also corresponds to the moDC subpopulation. Similarly, in all the datasets we used, the data contributors have clearly indicated that the DC cells included are tolDC, and these DC cells are human moDC subsets induced by clinical-grade dexamethasone and identified by sequencing analysis. Specific DC cell processing and identification can be seen in the description of the data contributors in each dataset. Information about these data sets has been briefly described in the ' 2.1 Data collection ' section of the manuscript ( line: 112-124 ).

**4. Other minor concerns: (1) Figures 1E and 1F appear to have an elliptical shape; some of the highlighting on the genes look weird in Fig1G. (2) Fig7 A-F are not necessary. (3) Fig7 H should be better organized to improve the readability. the current form is really hard for me to link its relationship with Fig7G, H;**

Re：Dear reviewer, thank you for your careful guidance. We have optimized the figure of Fig1 according to your suggestion, so that Fig1 E, F satisfies the expected circle instead of ellipse, and redraw the PPI network diagram of Fig1 G to improve its readability. Similarly, we have optimized and redrawn the original 7G, H, I, and J to improve their readability so that readers can better understand the connections between them. In addition, based on your suggestion, we adjusted Fig7, which is used to describe single cell data preprocessing, into Supplementary Fig 2 to make the manuscript context more concise and clear. Thank you again for your detailed guidance and valuable advice. Your guidance can make our manuscripts more perfect and provide more valuable help for the treatment of GC.

**Response to the letter of December 9,2025**

**1. Please include a legend for figure 7.**

Re : Dear editor, thank you for your valuable comments. We have added legends to Figure 7 in the manuscript to better explain the content of the diagram ( lines 567-569 of the manuscript ). It is hoped that this can improve readers ' understanding of the results. Thank you again for your attention and suggestions for our work, which is very helpful for us to improve the manuscript.

**2.Please upload a copy of Figure 8 which you refer to in your text on page 26. Or if the figure is no longer to be included as part of the submission please remove all reference to it within the text.**

Re : Dear editor, thank you for pointing out this problem. It should be noted that the legend of Fig 8 in the manuscript corresponds to Figure 7 of the current manuscript. In the revised manuscript, we have uniformly corrected the ' Fig 8 ' mentioned in the original text to ' Fig 7 ', and the legend has been updated synchronously to ensure that the text is consistent with the chart number. We apologize for the confusion. Thank you again for the reviewer 's careful review and valuable comments.

**3.We notice that your supplementary figures are uploaded with the file type 'Figure'. Please amend the file type to 'Supporting Information'. Please ensure that each Supporting Information file has a legend listed in the manuscript after the references list.**

Re : Dear editor, thank you for your reminder. Based on your comments, we have corrected the file type of the supplementary material to ' Supporting Information ', and added the corresponding legend description to the list of manuscript references ( the relevant content is in line 1112-1134 of the manuscript ). We apologize for the previous irregularities. Thank you again for your careful review and valuable suggestions.

**Response to the letter of December 18,2025**

**1. We note that there is identifying data in the Supporting Information file <Supplementary Table 2.xlsx, Supplementary Table 3.xlsx>. Due to the inclusion of these potentially identifying data, we have removed this file from your file inventory. Prior to sharing human research participant data, authors should consult with an ethics committee to ensure data are shared in accordance with participant consent and all applicable local laws.**

**Data sharing should never compromise participant privacy. It is therefore not appropriate to publicly share personally identifiable data on human research participants. The following are examples of data that should not be shared:**

**- Name, initials, physical address**

**- Ages more specific than whole numbers**

**- Internet protocol (IP) address**

**- Specific dates (birth dates, death dates, examination dates, etc.)**

**- Contact information such as phone number or email address**

**- Location data**

**- ID numbers that seem specific (long numbers, include initials, titled “Hospital ID”) rather than random (small numbers in numerical order)**

**Data that are not directly identifying may also be inappropriate to share, as in combination they can become identifying. For example, data collected from a small group of participants, vulnerable populations, or private groups should not be shared if they involve indirect identifiers (such as sex, ethnicity, location, etc.) that may risk the identification of study participants.**

**Please remove or anonymize all personal information (<Column with ID numbers>), ensure that the data shared are in accordance with participant consent, and re-upload a fully anonymized data set. Please note that spreadsheet columns with personal information must be removed and not hidden as all hidden columns will appear in the published file.**

Re : Dear Editor : Thank you very much for your careful review and reminder. We have carefully checked the supporting information files you mentioned ( Supplementary Table 2.xlsx, Supplementary Table 3.xlsx ) and confirmed that they do not contain any personal information or clinical data of human study participants.

The contents of the document are as follows :

Supplementary Table 1 is a list of tolDC-related genes.

The contents of Supplementary Table 2 and Supplementary Table 3 are the results of Gene Ontology ( GO ) functional enrichment analysis and Kyoto Encyclopedia of Genes and Genomes ( KEGG ) pathway enrichment analysis, respectively. In these two tables :

It does not contain any direct or indirect identification information you listed, such as name, address, age, date, contact information, geographical location, hospital ID, etc.

The ' ID ' column in the table is not a patient or clinical identifier, but a unique public identifier assigned to each functional or pathway entry by the GO database or the KEGG database. These IDs are public biological annotation standard numbers and do not involve the privacy of any individual participant.

Therefore, these supporting information tables contain only public, non-personal bioinformatics analysis results, fully comply with the ethical norms of data sharing, and do not pose any risk to the privacy of research participants.

We understand and fully support the principle of protecting participants ' privacy. Thank you for your reminder to ensure data compliance. We have reconfirmed that all shared data meet the relevant ethical requirements and journal regulations.

**2. Can you please upload an additional copy of your revised manuscript that does not contain any tracked changes or highlighting as your main article file. This will be used in the production process if your manuscript is accepted. Please amend the file type for the file showing your changes to Revised Manuscript w/tracked changes.**

Re : Dear Editor : Thank you for your reminder. We have updated the file in the submission system according to your requirements : The final version of the manuscript that does not contain any revision marks and highlights is uploaded as a ' Manuscript '.

All revision traces and changes marked versions will be displayed and uploaded as ' Revised Manuscript with Tracked Changes '. Both versions have been correctly uploaded and confirmed the file type. Thank you for your guidance.
